# Public Opinions on Removing Disincentives and Introducing Incentives for Organ Donation: Proposing a European Research Agenda

**DOI:** 10.3389/ti.2024.12483

**Published:** 2024-04-03

**Authors:** Frederike Ambagtsheer, Eline Bunnik, Liset H. M. Pengel, Marlies EJ Reinders, Julio J. Elias, Nicola Lacetera, Mario Macis

**Affiliations:** ^1^ Department of Internal Medicine, Nephrology and Kidney Transplantation, Erasmus MC Transplant Institute, University Medical Center Rotterdam, Rotterdam, Netherlands; ^2^ Department of Medical Ethics, Philosophy and History of Medicine, Erasmus Medical Center, Rotterdam, Netherlands; ^3^ Erasmus MC Transplant Institute, University Medical Center Rotterdam, Rotterdam, Netherlands; ^4^ Department of Economics, School of Business, University of CEMA, Buenos Aires, Argentina; ^5^ University of Toronto, Toronto, ON, Canada; ^6^ Carey Business School, Johns Hopkins University, Baltimore, MD, United States

**Keywords:** ethics in transplantation, payments, organ donation, incentives, disincentives

## Abstract

The shortage of organs for transplantations is increasing in Europe as well as globally. Many initiatives to the organ shortage, such as opt-out systems for deceased donation and expanding living donation, have been insufficient to meet the rising demand for organs. In recurrent discussions on how to reduce organ shortage, financial incentives and removal of disincentives, have been proposed to stimulate living organ donation and increase the pool of available donor organs. It is important to understand not only the ethical acceptability of (dis)incentives for organ donation, but also its societal acceptance. In this review, we propose a research agenda to help guide future empirical studies on public preferences in Europe towards the removal of disincentives and introduction of incentives for organ donation. We first present a systematic literature review on public opinions concerning (financial) (dis)incentives for organ donation in European countries. Next, we describe the results of a randomized survey experiment conducted in the United States. This experiment is crucial because it suggests that societal support for incentivizing organ donation depends on the specific features and institutional design of the proposed incentive scheme. We conclude by proposing this experiment’s framework as a blueprint for European research on this topic.

## Graphical Abstract

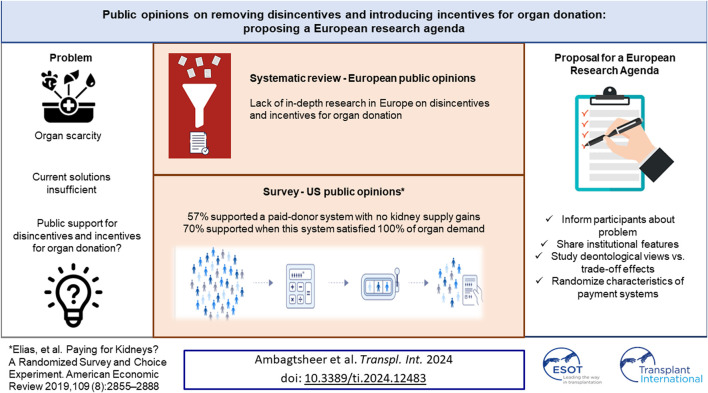

## Introduction

The shortage of organs for transplantations is longstanding and increasing in Europe as well as in the rest of the world. The policies that many European countries enacted, such as opt-out systems for deceased organ donation [1], have not been effective in filling the gap between the need and availability of organs [2]. Furthermore, significant disparities remain in deceased and living organ donation rates across Europe [3]. In 2022, there were still over 52,000 patients registered on wait lists in the European Union, of whom 42,000 needed a kidney transplant [4]. Roughly 100 million Europeans suffer from chronic kidney disease [5]. In 2022, in Europe, on average, 19 patients died every day while waiting for an organ transplant, and every hour, five new patients are added to transplant waitlists [6].

In recurrent discussions on how to address the plight of patients on waiting lists, monetary or non-monetary incentives have been suggested to stimulate organ donation and thus increase the pool of available donor organs. However, payments for organs are illegal in most countries. The ethical principle that “the human body and its parts shall not, as such, give rise to financial gain” [7] is broadly shared by governments, international organizations, and transplant societies [8, 9]. The prevailing position is that organ donation should be based on altruistic motivations and should be seen as a “gift” [10]. Although offering financial *incentives* to organ donors is prohibited, providing financial *compensation* is not [8]. Compensation, or reimbursement of the costs incurred by donors, including medical expenses, travel costs, and loss of income is intended to help to remove disincentives to living organ donation, but may not always suffice [11]. To encourage more people to donate, the use of monetary or non-monetary incentives might help.

The distinction between offering incentives and removing disincentives is unclear, however. The Nuffield Council on Bioethics describes a “(dis)incentive continuum” that ranges from “recompense” to “purchase,” or from reimbursement for incurred losses to direct payment in exchange for organs [12]. The American Society of Transplantation and the American Society of Transplant Surgeons similarly identify a spectrum of policy options, which they describe as an “arc of change” that should begin with removing disincentives that obstruct the organ donation process [13]. In-between compensating and paying, there are various possible forms of non-financial and indirect financial rewards [14], including granting donors priority positions on waiting lists and waiving donor health insurance premiums for certain amounts of time [15]. Some of these rewards may be compatible with the ethical principle that living organ donation should be “financially neutral to the donor” [16].

Throughout the years, various policy proposals suggesting different reward systems for deceased and living organ donation have been proposed [15, 17–22]. In Netherlands in 2007, for example, the Centre for Ethics and Health, a partnership of the Dutch Health Council and the Council for Public Health and Society, recommended the introduction of financial incentives for deceased and living organ donations to the Dutch government [15]. There have been similar proposals in the United States of America (United States), China, and Singapore [23–26]. Iran is currently the only country that allows payments for living kidney donation [27]. In most proposals for reward systems for living kidney donation, a national regulatory body would regulate the process, the healthcare system (not the recipient) would make the payments, and allocation would be based on medical need [15, 28]. Although the consequences of such a model will need to be monitored, its features may allay many ethical objections towards paying donors [15, 29]. Yet, there remains considerable opposition to the implementation of these proposals [30–32].

In the context of regularly resurfacing discussions on the legalization of incentives for organ donors, it is important to understand not only its ethical acceptability, but also its societal acceptance. In liberal democracies, public policies should ideally align with citizens’ moral perspectives and be upheld by stakeholders. On the one hand, given the widespread ethos that donation should be a gift, one might expect limited societal acceptance of (financial) incentives. On the other hand, markets that are assumed to be controversial or that a—in some countries—illegal, do not always elicit public repugnance [33]. It is thus crucial to approach this topic with nuance, as the debate surrounding payments for organs is often framed in black-or-white terms [34–36]. For instance, proposals for the introduction of incentives are often unduly equated with proposals for a free market for human body parts. Because, there are potentially numerous policy options for paid donation [20–22, 37], a more balanced consideration of public perspectives, ethical concerns and possible outcomes is warranted [38].

In this paper, we propose a research agenda to help guide future empirical studies on public preferences in Europe towards the removal of disincentives and introduction of incentives for organ donation. While our focus is on Europe, our considerations are also suitable for other regions. We include both deceased and living organ donation, but concentrate particularly on living kidney donation, consistent with most studies and policy proposals [15, 17–20]. In support of our objective, we present a systematic literature review on public opinions concerning (financial) (dis)incentives for organ donation in European countries. We do not only present the outcomes of these studies, but also critically discuss the nature and socio-demographic characteristics of the samples in these studies, the methodology used, and what questions these studies can and cannot answer. Next, we describe the results of a randomized survey experiment conducted in the United States in 2019 by Elias et al. [39]. This experiment is crucial because it suggests that societal support for incentivizing organ donation depends on the specific features and institutional design of the proposed incentive scheme. We conclude by proposing this experiment’s framework as a blueprint for European research on this topic.

## (Dis)Incentives for Organ Donation in Europe: Results of a Systematic Literature Search

Hoeyer et al. were the first to systematically synthesize studies on public attitudes towards financial incentives for organ donation [40]. Although their objective was to identify global trends in public opinions on financial incentives, they underscored the methodological challenges in comparing and aggregating studies due to variations in methods, contexts, and respondent selection. They also emphasized the marked differences in public opinions across these studies [40]. In their analysis of 23 studies from various countries across the globe, they observed, amongst others, a greater acceptance of financial incentives for organ donation in the United States and in the United Kingdom (UK), compared to other countries. In Central European countries (i.e., Germany, Austria, Switzerland, Netherlands) they observed minimal support for direct payments but a moderate acceptance of indirect benefits [40].

For our review, which focused exclusively on studies in European countries, we identified studies that focused on public opinions in Europe published after Hoeyer et al.’s research.

## Methods

### Inclusion and Exclusion Criteria

We included studies presenting empirical data on opinions regarding financial (dis)incentives from the European general public, including subgroup such as students, medical professionals, patients and donors. Financial disincentives included reimbursement of healthcare expenses, and financial incentives included free healthcare insurance for living donors and cash rewards for families of deceased donors. Studies presenting international opinions were included as long as results from European samples could be extracted. We excluded congress abstracts and studies published in languages other than English.

### Bibliographic Search

We conducted a systematic literature search to identify studies that reported European public opinions on financial (dis)incentives for living or deceased donor organ donation. An information specialist helped develop detailed bibliographic searches consisting of a combination of Medical Subject Heading (MeSH) terms and keywords for Medline, Embase and Psychinfo to identify studies that were published since the published literature review by Hoeyer et al. [40] i.e., from January 1, 2012 until April 20, 2023 (Appendix 1).

### Study Selection, Data Extraction and Analysis

**FIGURE 1:**
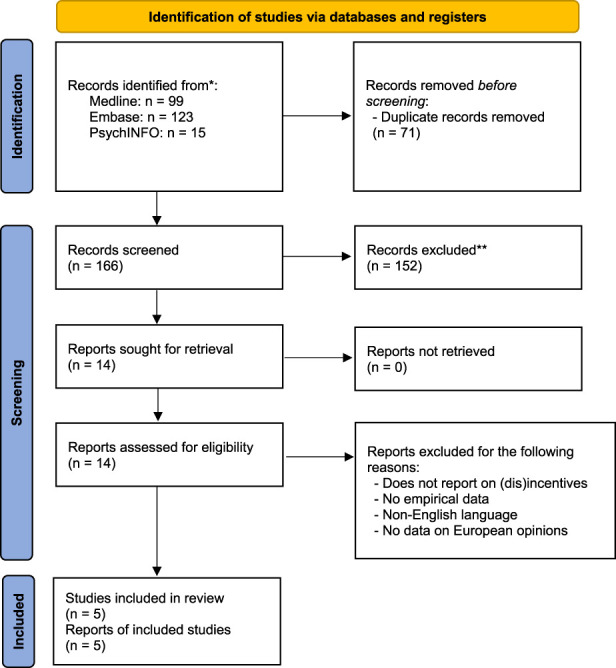
PRISMA flow diagram showing the identification, selection and inclusion of studies.

We merged the search results from the three bibliographic databases into a single EndNote database. Two reviewers (EMB and LHMP) independently screened the abstracts and titles, which was followed by full text review of potentially eligible studies. We resolved discrepancies between reviewers at any stage of this process by discussion and consultation with a third reviewer (FA). Figure 1 displays a flow diagram of the selection process according to the Preferred Reporting Items for Systematic Reviews and Meta-Analyses (PRISMA) [41]. One reviewer (FA) extracted the following data from the studies: year of publication, country, study design, sampling method, number of participants, participant demographics, overall objective of the study, questions on financial incentives and summary of findings. A second reviewer (LHMP) verified these data. Corresponding authors of the studies were contacted in case of missing data. We then summarized the studies in the form of a narrative review.

## Results

### Characteristics of Included Studies

Our bibliographic search identified 166 unique references of which five studies met our criteria for inclusion (Figure 1) [42–46]. There were four survey studies and one study reporting semi-structured interviews. Only Ghahramani et al. exclusively addressed financial incentives for organ donation as the main focus of their study [46]. The other four studies asked only few (between one and four) questions on financial incentives, which formed part of a larger survey or interview-study that addressed opinions on organ donation and transplantation more generally. For example, in their survey exploring public attitudes towards organ donation in Denmark, Nordfalk et al. only included two questions/statements, asking respondents whether *“[I]t would be fair if donors or relatives received compensation for any potential expenses in relation to the donation”* [44].

Public opinions included opinions from the general population, students, nephrologists and patients with end-stage kidney diseases (ESKD) who had publicly solicited a living kidney donor. Studies were conducted in Romania, Germany, Denmark and Netherlands. Ghahramani et al. [46] reported opinions of Eastern and Western European transplant nephrologists but did not specify the countries. One study reported on living donation [45], three studies reported on living and deceased donation [43, 44, 46] and one study did not specify whether the question related to living or deceased donation [42]. The corresponding author of the latter study [42] was contacted with the request to provide the question on financial compensation but no reply was received.

### Summary of Findings

The data in the studies were heterogeneous in terms of the characteristics of the study population, the country, and the framing of the questions regarding financial incentives (Table 1). Below we provide a narrative summary of the data, separately between living and deceased kidney donation.

**TABLE 1:** Characteristics of the included studies.

Author (year), country, living/deceased donation	Methodology, sampling method	Background (n), age, gender and ethnicity	Education, socio-occupational status, religion	Questions on financial incentives	Summary of findings
Bacușcă (2022), Romania	Survey	440 city residents	*Education*	Unclear how the question on financial compensation was framed	44.5% of respondents supported financial compensation, while 38.9% rejected financial compensation
		*Age* mean 43.5 y	33% higher education, 42% high school, 16% vocational, 7% elementary		
		*Gender (M:F)*	*Socio-occupational status*		
Unclear whether living or deceased donation	A 3-stage probability sampling technique to choose a representative sample of city residents	50%: 50%	44% employed, 21% retired, 17% students, 16% freelancers, 7% housekeepers, 5% unemployed		
		*Ethnicity*	*Religion*		
		NR	Christian (98%)		
Ghahramani (2013), Eastern and Western Europe	Survey	230 Eastern and Western European transplant nephrologists. They were part of a larger sample of a total of 1,280 international nephrologists	*Education*	Four questions explored opinions around the following topics	Q1) Nephrologists from Eastern Europe were more likely to agree with health insurance for donors compared to nephrologists from Canada/United States but there was no difference between nephrologists from Canada/United States and Western Europe
Living and deceased donation	A database of email addresses was created by an online search method which was supplemented by lists from national and regional nephrology societies	*Age*	NR	Q1) Free lifelong health insurance for living donors	Q2) Nephrologists from Western Europe were less likely to favor direct financial compensation for living donation compared to nephrologists from Canada/United States but there was no difference between nephrologists from Canada/United States and Eastern Europe
		60% ≤ 50 years; 40% > 50 years (all 1,280 respondents)	*Socio-occupational status*	Q2) Some form of (direct) financial compensation for living donors	Q3) Nephrologists from Western Europe were less likely to agree with financial rewards to living-related or living-unrelated donors compared with nephrologists from Canada/United States but there was no difference between nephrologists from Canada/United States and Eastern Europe
		*Gender (M:F)*	Transplant nephrologists	Q3) Financial rewards for living related and unrelated donors	Q4) Nephrologists from Western Europe were less likely to agree with providing financial rewards to families of deceased donors compared with nephrologists from Canada/United States but there was no difference between nephrologists from Canada/United States and Eastern Europe
		72%: 28% (all 1,280 respondents)	*Religion*	Q4) Financial rewards for families of deceased donors	
		*Ethnicity*	NR		
		NR			
Inthorn (2014), Germany	Survey	755 students (466 students of medicine and 289 students of economics)	*Education*	Four questions explored opinions on commercialization and compensation for organ donation	LOD: Only 5% of medical students and 9% of economics students were in favor of allowing to sell one’s organs for money. The majority (73%) believed that a living donor should receive cheaper or free follow-up treatment, while 9% felt that a living donor should receive free life insurance from the state. Overall, students favored removing disincentives, e.g., compensation for health and surgery related costs, or models of reciprocity (living donors receive benefits when they need an organ themselves) over monetary ‘incentives’, such as cash rewards. Still, only 45% of students felt that living donors should be compensated for the related health expenses
		*Age*			
		0–19years: 14%	University students	Q1) Financial incentives for living organ donors	
		20–24years: 63%			
		25–29years: 20%	*Socio-occupational status*	Q2) Statements on financial compensation	
Living and deceased donation	Students were asked to participate after compulsory classes	≥30years: 3%			DOD: Although both groups of students tended to reject financial models, the number of students favoring financial incentives was higher among economics students compared to medical students in four out of six questions. The authors state that there was a relatively high number of students who were undecided but these data were not shown
		*Gender (M:F)*	University students	Q3) One-off payments for living donors	
		48%: 52%			
		*Ethnicity*	*Religion*	Q4) Economic incentives following *postmortem* donation	
		NR	NR		
Nordfalk (2016), Denmark	Survey	1,195 Danish citizens	*Education*	Respondents were asked to rate their agreement with the following statements	Only 6% of citizens found it acceptable to use money as a motivation for donating organs and a slight majority (52.7%) agreed to compensate expenses related to the donation
		*Age*	Secondary: 40%		
		Mean 50years (range: 18–102)	Post-secondary: 33%		
		*Gender (M:F)*	Short-cycle tertiary: 5%	1) “It should be possible to motivate donors or relatives of potential donors with money, to make them donate organs”	For both of these questions, women tended to disagree more with the statements than men (*p* < 0.05)
		49%: 51%	Bachelor: 15%		
Living and deceased donation		*Ethnicity*	Master: 7%		
		NR	*Socio-occupational status*	2) It would be fair if donors or relatives received compensation for any potential expenses in relation to the donation”	The data showed a clear difference between attitudes to money used as incentives and as compensation
			NR		
			*Religion*		
			Christian protestantism: 21%; Muslim: 2%; Other: 4%; not religious: 73%		
Pronk (2018), Netherlands	Semi-structured interviews	20 Dutch patients with end-stage renal disease who had publicly solicited a living kidney donor	*Education*	Patients were asked the following questions	The majority of participants disapproved of buying a kidney, because they feared blackmailing, believed this would be unfair to patients who do not have the money to buy a kidney, or because they did not want to violate the law. They also believed it would be too risky to be transplanted with a traded kidney and did not want to benefit from someone else’s poverty
		*Age*Mean 46years (range: 26–74)	Primary or secondary education: 35%		
			Further education: 65%	Q1) Do you believe that a public appeal for a kidney donor attracts people who want to get something in return for their kidney? For example, financial or social. Would you object to that?	Some participants reported that they would buy a kidney if they would have the means to do so or if their medical situation became more urgent, implying that they perceived public solicitation as a step that can be taken prior to exploring paid donation. Almost all participants received offers of a kidney in return for money or payment in kind (such as employment, residency, or sexual favors). Participants also received offers from prisoners who wanted to do something good for another person
Living donation	Google, Facebook and Twitter were searched to identify Dutch kidney patients and their representatives who publicly solicited a living kidney donor. Eligible patients were invited by email, telephone or social media	*Gender (M:F)*	*Socio-occupational status*		
		60%: 40%	NR	Q2) In general, do you believe that in the Netherlands, something could or should be offered to donors, some kind of compensation or financial reward? What do you think of that and what kind of compensation do you have in mind?	Offers for payment (in kind) appalled participants and were ignored or turned down. They wanted a kidney to be an unconditional gift from a donor
		*Ethnicity*	*Religion*		
		‘Dutch’	NR		

NR, not reported; DOD, deceased organ donation; LOD, living organ donation.

### Living Kidney Donation

Four studies surveyed public opinions regarding financial (dis)incentives for living kidney donation [43–46]. Overall, more participants tended to agree than disagree with reimbursing the costs incurred by the donation and/or allowing more indirect rewards, such as a free life-long health insurance or cheaper or free follow-up treatments [43–45]. Only a very small percentage would agree with direct financial rewards, such as cash payments [43–45].

Participants in the study by Pronk et al. also highlighted perceived risks for recipients of being transplanted with a traded kidney and an unease among recipients with benefiting from other people’s poverty [45]. Most participants who had experience with public solicitation of living donors had received offers of kidneys in return for money or payment in kind, for example, employment or residency. Payments in kind were considered unacceptable to the participants and were turned down. Respondents considered public solicitation as a first step in finding a kidney donor before exploring paid donation, which they would consider if they had the means to pay a donor or if their medical condition became more urgent [45].

Ghahramani et al. [41] compared opinions of Eastern and Western European nephrologists with opinions of nephrologists from non-European countries (i.e., Canada and the United States). Eastern European nephrologists were more likely to agree with providing free life-long health insurance for living donors compared to nephrologists from non-European countries. Western European nephrologists were less likely to favor direct financial payments or rewards compared to nephrologists from non-European countries, whilst no differences were found between nephrologists from non-European countries and Eastern Europe.

### Deceased Kidney Donation

Three studies reported opinions regarding financial incentives for deceased donation [43, 44, 46]. Financial models for deceased organ donation based on incentives were rejected by most participants [43, 44]. Ghahramani et al. reported that nephrologists from Western Europe were less likely to agree with providing financial rewards to families of deceased donors compared to nephrologists from Eastern Europe and other geographic reasons [46].

## Paying for Kidneys? Results of a Randomized Survey and Choice Experiment in the United States

In 2019, Elias et al. published the findings of a randomized survey experiment concerning the preferences of American citizens for paying living kidney donors [39]. The study assessed whether attitudes toward a paid-donor system depend on its possible effects on the number of transplants (i.e., lives saved), or whether they reflect deontological views or “sacred values.” Moreover, the survey investigated whether and how preferences respond to different institutional features of a hypothetical paid-donor system, the moral foundations of preferences for paid-donor systems, and the extent to which attitudes are heterogeneous in the population. The study’s sample included nearly 2,700 American residents, stratified to match the United States population across various demographics.

The study’s design included the random assignment of respondents to consider one hypothetical paid-donor kidney procurement and allocation system, asking them to view it as an alternative to the current system in which kidney donors do not receive payment. There were eight possible paid-donor systems, which were the combination of the following characteristics: the type of payment (direct cash or non-cash, like contributions to college or retirement funds), the payment amount ($30,000 or $100,000), and the entity responsible for payment (either the organ recipient or a public agency). Subsequently, each respondent made five decisions about expressing support either for the proposed donor payment system or to maintain the existing, unpaid-donor system. In all five choice situations, the characteristics of the alternative system remained the same, with the only difference being the kidney supply gains, i.e., the number of additional transplants that participants were asked to assume the paid-donor system would produce in each scenario. The survey presented the five scenarios in a sequence, starting from no increase in organ donations and progressing to an increase in donations sufficient to completely eliminate the waiting list [39].

There was wide heterogeneity in preferences and strong polarization of attitudes among respondents, with large proportions of respondents either in favor of or against paying kidney donors regardless of the size of hypothesized kidney supply gains. However, the study found that support for paying donors becomes stronger when the projected increase in the number of transplants is higher. On average, 57% of respondents supported a paid-donor system with no kidney supply gains, and about 70% supported a paid-donor system when the system satisfied 100% of the demand for organs. Thus, a considerable proportion of respondents have “tradeoff-sensitive” attitudes, because their views depended on the number of additional transplants that could be obtained through a paid-donor system. When there was a sufficient increase in the availability of kidneys, these individuals were more inclined to support the legalization of a paid-donor system and had fewer ethical concerns [39].

**FIGURE 2:**
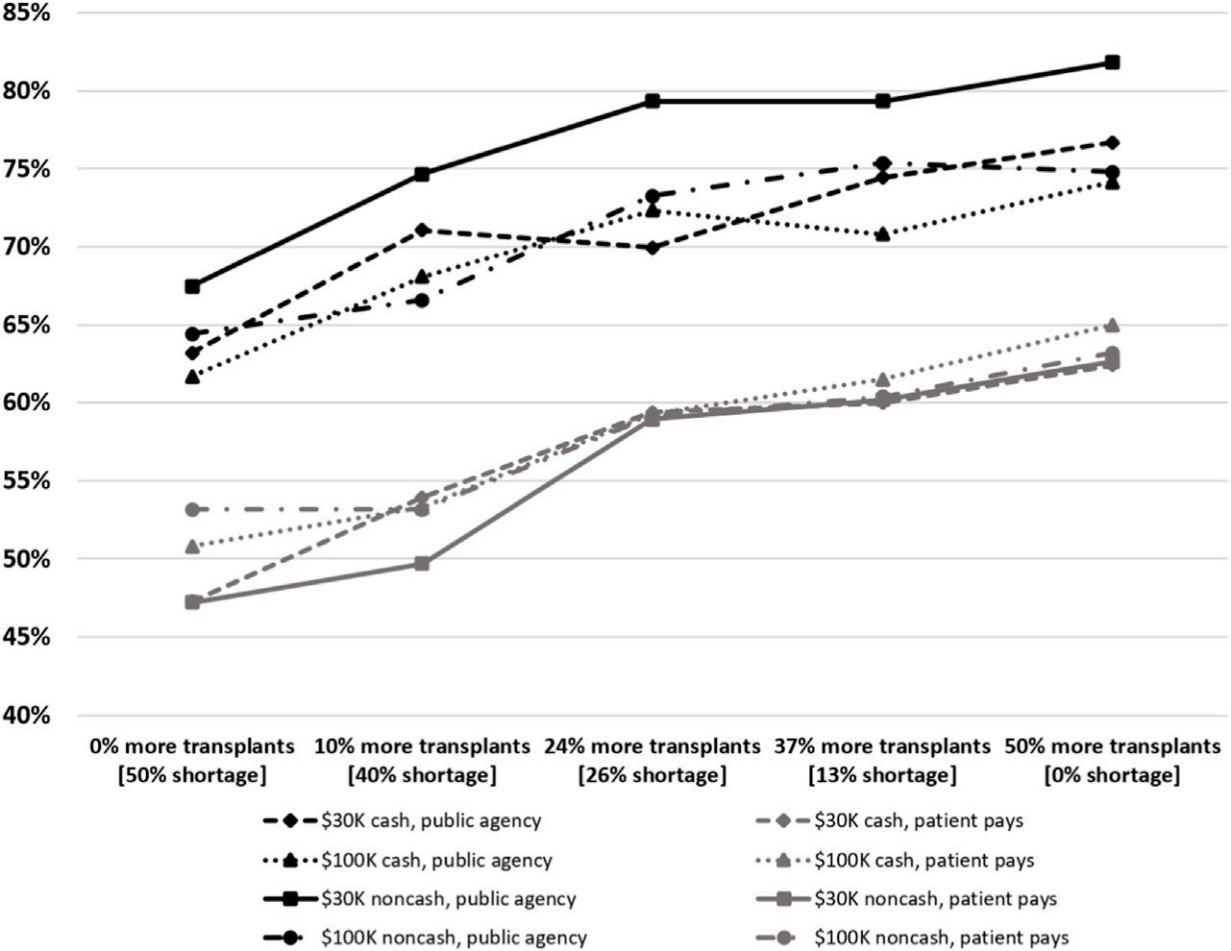
Support for Paid-Donor Systems. Notes: The figure reports the percentage of respondents in favor of compensating kidney donors, by payer (public agency or patient), amount of compensation ($30 K or $100 K), nature of compensation (cash or noncash), and hypothesized kidney supply level. We assessed how much of the annual demand for transplants, not the waiting list, would be affected by the increase in the number of transplants. Source: [39]. (Copyright American Economic Association; reproduced with permission of the American Economic Review).

Further, the level of support for paid-donor systems varied substantially according to the identity of the payer. Specifically, a large share of respondents opposed the private transactions where the kidney recipient would pay the donor (either directly or through their insurance). However, respondents showed much stronger support for procurement and allocation systems in which a public agency pays kidney donors and allocates organs using a mechanism similar to the current algorithm that distributes deceased donor organs based on medical urgency, blood and tissue match, time on the waiting list, etc. This finding indicates that there is a difference in opinions vis-à-vis “paying donors” and “allowing patients to purchase an organ.” Opposition to the latter is very strong, whereas a large proportion of respondents supports paying organ donors when this is performed by a public agency that allocates the resulting organs fairly (i.e., not based on the patient’s ability to pay). The nature and amount of payment did not have a large effect on support for paying donors. However, the paid-donor system with the highest support (more than 80% of respondents) was the one where a public agency provides donors with $30,000 noncash compensation (e.g., in the form of contribution to a retirement account) (Figure 2).

The study also assessed whether respondents’ attitudes were based on deontological or “sacred” values toward paying living kidney donors. The authors asked participants to express their moral judgments about both the current system and the paid-donor system to which they were assigned, at each hypothesized organ supply level. The six ethical principles considered—autonomy of choice, undue influence, exploitation of the donor, fairness to the donor, fairness to the patient, and human dignity—accounted for a substantial share of the variance in support for paid donor systems. Moral judgments were especially affected by the identity of the payer and the nature of compensation. In particular, respondents viewed non-cash payments and payments by a public agency as more ethical than cash payments and payments by the organ recipient, and were most concerned about the fairness of organ allocation, which was the primary reason for their opposition to systems that involved payments made by the organ recipient [39].

## Conclusion: A Proposal for a Research Agenda on (Dis)incentives for Organ Donation in Europe

Our systematic literature review suggests that there is relatively little public support in Europe for financial incentives—especially cash payments—for organ donation, and some public support for removing disincentives. Yet, only five studies on public opinions have been conducted over the last decade in Europe. Furthermore, the majority of these studies did not focus on (dis)incentives as their main topic, but incorporated only a few questions on the issue as part of a larger investigation on public opinions on organ donation. Additionally, the questions tended to be too generic to be truly informative, as they neither specified the relevant characteristics of the proposed policies nor addressed the expected effects on organ supply. Thus, the research body in Europe on this subject is limited both in volume and methodologically, and does not allow for an in-depth and conclusive empirical assessment of the degree of public support for (dis)incentives for organ donation.

We posit that the topic of (dis)incentives for organ donation should be the central focus of in-depth studies that incorporate the various features of paid donation schemes, their implications for the donor organ supply and the nuanced moral and practical considerations that underlie them. Elias et al. demonstrate the importance of including at least four critical features when studying the delicate and complex topic of (dis)incentives for organ donation amongst the general public [39]. First, participants should be informed about the problem (e.g., number of waitlisted patients, waiting times, etc.), its implications (e.g., patient mortality, healthcare costs, etc.) and possible alternatives (e.g., paid-donor systems). Second, the various institutional characteristics that may underlie different paid-donor systems should be described. It is critical to recognize that the way a system is structured can greatly influence its public acceptance. For instance, the ethical considerations associated with a free-market exchange between prospective donors and recipients stand in stark contrast to the ethical considerations related to a government-controlled system that offers non-financial rewards for deceased donation or living kidney donation and that allocates organs based on medical need. This distinction is vital, as it underscores the necessity to meticulously define and communicate the relevant features of any proposed policy, ensuring that respondents fully grasp the implications and nuances of each system. Public opinions may also vary according to the type of incentive or disincentive that is offered. Third, studies of this topic should explore whether public opinions are influenced by the possible effects of paid donation systems on the number of transplants (i.e., gains in patients’ life expectancy). It is an empirical question, and not an assumption, that the opposition to compensation and payments responds to some “sacred values” and is not amenable to the considerations of other socially relevant outcomes. Finally, adding experimental manipulation to the design of surveys is paramount for determining causality. By randomly varying the characteristics of the institutional design, researchers can directly assess how each feature impacts the acceptability of specific paid-donor systems, both from a moral and practical standpoint. This approach offers a more precise understanding of public attitudes towards the intricate balance between ethical concerns and pragmatic needs.

In light of calls for trials to experiment with payments for both living [17, 20, 21] –and deceased donation [47–49], our proposed research agenda can generate the needed evidence to evaluate the acceptability in the general population towards allowing payments for deceased and living organ donation.

In proposing a European research agenda, we call for the integration of these critical features into future empirical studies of this topic (Table 2). Such an approach will delve deeply into the intricate perceptions surrounding paid donor schemes. Moreover, it will clarify the specific conditions and frameworks under which general publics might deem such schemes acceptable. This information can guide law- and policymakers and other stakeholders in developing policy proposals on this topic. Erasmus MC’s Transplant Institute recently received funding from the Dutch Research Council that allows us to survey public opinions across three European countries, namely, Germany, Netherlands and Spain, while incorporating the aforementioned critical features [50, 51].

**TABLE 2:** A proposal for a research agenda on (dis)incentives for organ donation in Europe.

Introducing four critical features for future studies on opinions regarding paid donation schemes based on Elias et al. [39]	
1. Informing participants about the problem and about alternative solutions to the problem	Include, at a minimum, the number of waitlisted patients, waiting times, patient mortality rates, healthcare costs and alternatives (e.g., paid-donor systems)
2. Institutional characteristics	Government controlled payments; free market exchanges; organ allocation criteria; payment amount; type of monetary and non-monetary incentives; removal of disincentives
3. Deontological views vs. trade-off effects	Questions on sacred values versus expected trade-offs (e.g., higher patients’ lives expectancy, shorter transplant lists), results of payment schemes; assess moral and practical views
4. Experimental methods; randomizing characteristics of payment systems	Assess how each feature impacts the acceptability of specific paid-donor systems

Over the last three decades, numerous moral concerns have been raised against allowing payments for organs [52–54], with many proposing various specific market designs to attenuate those concerns [17, 20, 21]. Additionally, there is an ongoing debate about the effectiveness of a compensation system in terms of its impact on the number of transplants [52, 55, 56]. The contribution of our proposed research direction lies in causally estimating how the specific design of the system and its effectiveness could influence the general population’s acceptance of the system. Our aim is to provide new insights into studying the multifaceted perspectives of the European public on (dis)incentives on organ donation. Furthermore, we hope that our proposed methodology becomes a reference for other research teams. Such an approach is needed to comprehensively address and understand the complexities surrounding (dis)incentives for organ donation and to explore policy options to increase the supply of organs.

## Data Availability

The original contributions presented in the study are included in the article/Supplementary Material, further inquiries can be directed to the corresponding author.
