# Supplementary material for: Public Opinions on Removing Disincentives and Introducing Incentives for Organ Donation: Proposing a European Research Agenda
Source: Transpl Int. 2024 Apr 3;37:12483. doi: 10.3389/ti.2024.12483 (PMC11027084; doi:10.3389/ti.2024.12483)
Supplement: Supplementary file 1 [file DataSheet1.docx]

**Appendix 1 – Detailed Search Strategy**

| **Database searched** | **Platform** | **Years of coverage** | **Records** | **Records after duplicates removed** |
| --- | --- | --- | --- | --- |
| Medline ALL | Ovid | 1946 - Present | 99 | 99 |
| Embase | Embase.com | 1971 - Present | 123 | 64 |
| PsycINFO | Ovid | 1806 - Present | 15 | 3 |
| **Total** | | | **237** | **166** |

**Medline**

("Tissue Donors"/ OR "Living Donors"/ OR "Organ Transplantation"/ OR "Kidney Transplantation"/ OR "Liver Transplantation"/ OR ((organ* OR kidney* OR renal OR liver* OR hepatic* OR living OR non-related* OR nonrelated*) ADJ3 (donor* OR donation* OR transplant*)).ab,ti,kf. OR ((Commerc* OR paid OR financial* OR incentive* OR sale*) AND (donor* OR donation* OR organ* OR kidney* OR liver*)).ti.) **AND** ("Reward"/ OR "Remuneration"/ OR "Compensation and Redress"/ OR (((economic* OR financ* OR montar*) ADJ3 (motivation*)) OR incentive* OR sale* OR reward* OR remuneration* OR compensat* OR reimburse* OR Commerc* OR paid OR payment* OR money*).ab,ti,kf.) **AND** ("Public Opinion"/ OR "Attitude"/ OR (attitude* OR ((public OR general* OR population*) ADJ6 (opinion* OR sentiment* OR mind OR survey* OR perspective* OR Questionnaire* OR Survey*))).ab,ti,kf. OR (Questionnaire* OR Survey*).ti.) NOT ((decease* OR cadaver* OR dead).ti.) **AND** (exp "Europe"/ OR (Europe* OR Albania* OR Andorr* OR Armenia* OR Austria* OR Azerbaija* OR Balkan* OR Baltic* OR Estonia* OR Latvia* OR Lithuania* OR Belarus* OR Belgium* OR Benelux* OR Bosnia* OR Herzegovina* OR Bulgaria* OR Channel-Islands* OR Croatia* OR Czech* OR Denmark* OR Danish* OR England* OR Faroe* OR Finland* OR Finnish* OR France* OR French* OR Georgia* OR German* OR Gibraltar* OR Great-Brit* OR Greece* OR Greek* OR Hungar* OR Iceland* OR Ireland* OR Irish* OR Isle-of-Man* OR Ital* OR Kosov* OR Liechtenstein* OR Luxembourg* OR Malt* OR Moldova* OR Monaco* OR Montenegr* OR Netherland* OR Dutch* OR Norway* OR Norweg* OR Poland* OR Polish* OR Portug* OR Macedonia* OR Romania* OR Russian* OR San-Marin* OR Scandinavia* OR Scotland* OR Scottish* OR Serbia* OR Slovak* OR Slovenia* OR Spain* OR Spanish* OR Sweden* OR Swedish* OR Switzerland* OR Swiss* OR Ukraine* OR United-Kingdom* OR Vatican* OR Wales* OR Welsh*).ab,ti,kf.)

**Embase**

('organ donor'/de OR 'living donor'/de OR donor/mj OR 'kidney donor'/de OR 'liver donor'/de OR 'organ transplantation'/de OR 'kidney transplantation'/de OR 'liver transplantation'/de OR ((organ* OR kidney* OR renal OR liver* OR hepatic* OR living OR non-related* OR nonrelated*) NEAR/3 (donor* OR donation* OR transplant*)):ab,ti,kw OR ((Commerc* OR paid OR financial* OR incentive* OR sale*) AND (donor* OR donation* OR organ* OR kidney* OR liver*)):ti) AND ('economic incentive'/de OR 'monetary reward'/de OR reward/de OR 'remuneration'/de OR compensation/de OR reimbursement/de OR 'commercial phenomena'/de OR market/de OR (((economic* OR financ* OR montar*) NEAR/3 (motivation*)) OR incentive* OR sale* OR reward* OR remuneration* OR compensat* OR reimburse* OR Commerc* OR paid OR payment* OR money*):Ab,ti,kw) AND ('public opinion'/de OR attitude/de OR (attitude* OR ((public OR general* OR population*) NEAR/6 (opinion* OR sentiment* OR mind OR survey* OR perspective* OR Questionnaire* OR Survey*))):ab,ti,kw OR (Questionnaire* OR Survey*):ti) NOT ('deceased donor'/exp/mj OR (decease* OR cadaver* OR dead):ti) AND (Europe/exp OR (Europe* OR Albania* OR Andorr* OR Armenia* OR Austria* OR Azerbaija* OR Balkan* OR Baltic* OR Estonia* OR Latvia* OR Lithuania* OR Belarus* OR Belgium* OR Benelux* OR Bosnia* OR Herzegovina* OR Bulgaria* OR Channel-Islands* OR Croatia* OR Czech* OR Denmark* OR Danish* OR England* OR Faroe* OR Finland* OR Finnish* OR France* OR French* OR Georgia* OR German* OR Gibraltar* OR Great-Brit* OR Greece* OR Greek* OR Hungar* OR Iceland* OR Ireland* OR Irish* OR Isle-of-Man* OR Ital* OR Kosov* OR Liechtenstein* OR Luxembourg* OR Malt* OR Moldova* OR Monaco* OR Montenegr* OR Netherland* OR Dutch* OR Norway* OR Norweg* OR Poland* OR Polish* OR Portug* OR Macedonia* OR Romania* OR Russian* OR San-Marin* OR Scandinavia* OR Scotland* OR Scottish* OR Serbia* OR Slovak* OR Slovenia* OR Spain* OR Spanish* OR Sweden* OR Swedish* OR Switzerland* OR Swiss* OR Ukraine* OR United-Kingdom* OR Vatican* OR Wales* OR Welsh*):ab,ti,kw)

**PsychINFO**

(tissue donation/ OR organ transplantation/ OR ((organ* OR kidney* OR renal OR liver* OR hepatic* OR living OR non-related* OR nonrelated*) ADJ3 (donor* OR donation* OR transplant*)).ab,ti. OR ((Commerc* OR paid OR financial* OR incentive* OR sale*) AND (donor* OR donation* OR organ* OR kidney* OR liver*)).ti.) **AND** (exp rewards/ OR (((economic* OR financ* OR montar*) ADJ3 (motivation*)) OR incentive* OR sale* OR reward* OR remuneration* OR compensat* OR reimburse* OR Commerc* OR paid OR payment* OR money*).ab,ti.) **AND** (Public Opinion/ OR Attitudes/ OR (attitude* OR ((public OR general* OR population*) ADJ6 (opinion* OR sentiment* OR mind OR survey* OR perspective* OR Questionnaire* OR Survey*))).ab,ti. OR (Questionnaire* OR Survey*).ti.) **NOT** ((decease* OR cadaver* OR dead).ti.) **AND** ((Europe* OR Albania* OR Andorr* OR Armenia* OR Austria* OR Azerbaija* OR Balkan* OR Baltic* OR Estonia* OR Latvia* OR Lithuania* OR Belarus* OR Belgium* OR Benelux* OR Bosnia* OR Herzegovina* OR Bulgaria* OR Channel-Islands* OR Croatia* OR Czech* OR Denmark* OR Danish* OR England* OR Faroe* OR Finland* OR Finnish* OR France* OR French* OR Georgia* OR German* OR Gibraltar* OR Great-Brit* OR Greece* OR Greek* OR Hungar* OR Iceland* OR Ireland* OR Irish* OR Isle-of-Man* OR Ital* OR Kosov* OR Liechtenstein* OR Luxembourg* OR Malt* OR Moldova* OR Monaco* OR Montenegr* OR Netherland* OR Dutch* OR Norway* OR Norweg* OR Poland* OR Polish* OR Portug* OR Macedonia* OR Romania* OR Russian* OR San-Marin* OR Scandinavia* OR Scotland* OR Scottish* OR Serbia* OR Slovak* OR Slovenia* OR Spain* OR Spanish* OR Sweden* OR Swedish* OR Switzerland* OR Swiss* OR Ukraine* OR United-Kingdom* OR Vatican* OR Wales* OR Welsh*).ab,ti.)
